# mGem: Dentistry is strategically positioned yet underleveraged in the battle against antimicrobial resistance

**DOI:** 10.1128/mbio.00647-26

**Published:** 2026-08-10

**Authors:** Jonathon L. Baker, Ulf R. Dahle, Fernanda C. Petersen

**Affiliations:** 1 Department of Biomaterial and Biomedical Sciences, OHSU School of Dentistry 212162 , Portland, Oregon, USA; 2 Department of Biomedical Engineering, OHSU School of Medicine 89020, Portland, Oregon, USA; 3 Centre for Antimicrobial Resistance, Norwegian Institute of Public Health 25563, Oslo, Norway; 4 Institute of Oral Biology, University of Oslo 6305, Oslo, Norway; Georgia Institute of Technology, Atlanta, Georgia, USA

**Keywords:** dentistry, oral microbiome, antimicrobial resistance

## Abstract

Dentists account for roughly 10% of global antibiotic prescriptions and maintain a substantial patient contact footprint. As a result, dentistry is strategically positioned in the global response to the antimicrobial resistance (AMR) crisis, offering unique opportunities to impact antibiotic stewardship, AMR surveillance, and infection prevention. However, these opportunities are underleveraged because antibiotic stewardship is insufficiently emphasized in dental education curricula and competencies, and because dentistry is inconsistently integrated into AMR policy and national action plans. This disconnect perpetuates fragmentation in prescribing practices and weakens the alignment of dentistry with global One Health efforts. Furthermore, it leaves dentistry alienated from broader AMR research, funding, and educational frameworks. Specific integration of dentistry into AMR National Action Plans and implementation of unified, research-supported prescribing guidelines, targeted clinician education, and oral resistome surveillance could help transform dentistry from a blind spot to a fulcrum in the global AMR response.

## PERSPECTIVE

Antimicrobial resistance (AMR) is among the most significant global health threats of the 21^st^ century, compromising the effectiveness of antibiotics that underpin modern medicine, surgery, transplantation, and infection prevention (1–3). The World Health Organization (WHO) and the United Nations have called for coordinated international action through the Global Action Plan on AMR. In response, many countries have published AMR national action plans (NAPs), which typically blueprint antibiotic stewardship, surveillance, and infection prevention across a One Health framework that integrates the human, animal, and environmental health sectors (1–3). Despite these efforts, dentistry has remained inconsistently integrated into national and global AMR strategies. Consequently, dentistry is frequently excluded from coordinated AMR surveillance systems, stewardship infrastructure, educational initiatives, and targeted research funding. This omission represents a major missed opportunity. Dentists account for approximately 7%–10% of all antibiotic prescriptions in many high-income countries (4, 5). Beyond prescribing volume alone, dentistry occupies a unique clinical interface between primary care, surgery, and chronic disease prevention. Importantly, the oral cavity itself constitutes a large and dynamic reservoir of antimicrobial resistance genes, which may contribute to resistance dissemination beyond localized oral disease (6). In this perspective, we examine the systemic stewardship, governance, and policy failures that have limited dentistry’s integration into broader AMR responses. We conclude with proposed policy actions that could reposition dentistry from a peripheral stakeholder to a strategic partner within global One Health AMR initiatives. Note that while this article standardly employs the term “dentistry” in alignment with major governing bodies, the authors acknowledge that “dental medicine” is an increasingly preferred term in some settings, and the field should be viewed as an extension of systemic medicine, wherein oral health is inextricably linked to overall physiological well-being.

## ORAL RESISTOME AND ECOLOGICAL RELEVANCE

The oral cavity represents a uniquely dense and dynamic microbial ecosystem that harbors a diverse repertoire of antimicrobial resistance genes and may contribute to their dissemination within and beyond the host (6). Positioned at the intersection of the digestive and respiratory tracts, the oral cavity is part of a network of interconnected microbial ecosystems within the human host. Exposure of oral microbial communities to systemic antibiotics may exert collateral selective pressure across complex biofilm-associated populations, extending effects beyond the targeted pathogen. Dental plaque biofilms contain multispecies bacterial communities in close physical proximity, creating ideal conditions for horizontal gene transfer of antibiotic resistance genes through plasmids, transposons, bacteriophages, and other mobile genetic elements (6). Consequently, antibiotic exposure influences not only target pathogens but also surrounding commensal microbial communities, contributing to wider emergence, enrichment, and dissemination of AMR (3, 7). These ecological effects are increasingly recognized to have implications extending beyond oral disease. Resistant organisms and resistance determinants originating within the oral cavity can be transmitted within and between individuals or disseminated broadly within shared community and environmental ecosystems. Viewed through a One Health lens, the oral cavity should be considered an active interface in AMR ecology rather than an isolated compartment, reinforcing the importance of incorporating dentistry into coordinated AMR stewardship and surveillance frameworks.

## STEWARDSHIP FAILURES IN DENTAL PRACTICE

Multiple studies across several nations consistently demonstrated that dentists contribute substantially to outpatient antibiotic prescribing, with dentists in the USA writing roughly 25 million antibiotic prescriptions per year (4, 5, 7–9). Notably, 60%–80% of these prescriptions were inappropriate per current guidelines and clinical indication (10–14). While antibiotics are appropriate in select situations including spreading infection, sepsis, or prophylaxis for high-risk patients, definitive operative management should remain the cornerstone of treatment for most localized odontogenic infections (15). Common examples of overprescribing include prophylactic antibiotics before routine dental procedures in patients with prosthetic joint implants, unnecessary antibiotic use for localized endodontic infections that are amenable to definitive operative treatment, and prophylactic prescribing associated with uncomplicated extractions or implant placement. The factors driving this unnecessary antibiotic prescribing in dentistry are multifactorial and systemic. A major contributor is the fragmentation or inconsistency in existing prescribing guidelines, or even a lack of guidelines in some situations (16, 17). Among practitioners, this ambiguity leads to low confidence (under 50% in some cases) about whether antibiotics are really needed. In some health care systems, this drives defensive prescribing behavior which is often influenced by fear of litigation (18). Other drivers of unnecessary prescribing include limited access to urgent dental care, patient expectations, time pressures, diagnostic uncertainty, avoidance or delay of invasive procedures, outdated prescribing habits, and clinical inertia (7, 18–20). Awareness of updated guidelines and AMR NAPs remains low (~20%–25%) in many settings (21–23). These challenges are amplified in many low- and middle-income countries where over-the-counter antibiotic access and fragmented dental care systems complicate stewardship efforts (24–27). Effective antibiotic stewardship in dentistry will need to include timely operative treatment, accurate diagnosis, appropriate prophylaxis, and avoidance of unnecessary empiric antibiotic exposure.

Stewardship efforts are also hindered by surveillance gaps. Although many national AMR monitoring systems track community antibiotic usage, relatively few distinguish dental prescriptions in ways that support benchmarking, quality assessment, or guideline concordance evaluations (28). Improved integration of dental prescribing into national surveillance systems could substantially strengthen stewardship implementation. Linking prescriptions to diagnostic data would allow assessment of guideline adherence, while clinician feedback systems and peer benchmarking could similarly reduce unnecessary antibiotic use in dentistry (28, 29). Building on this, existing national primary care audit-and-feedback antimicrobial stewardship frameworks can, in principle, be structurally expanded and scaled up to encompass dental practitioners.

## GOVERNANCE AND POLICY FAILURES

**Fig 1:**
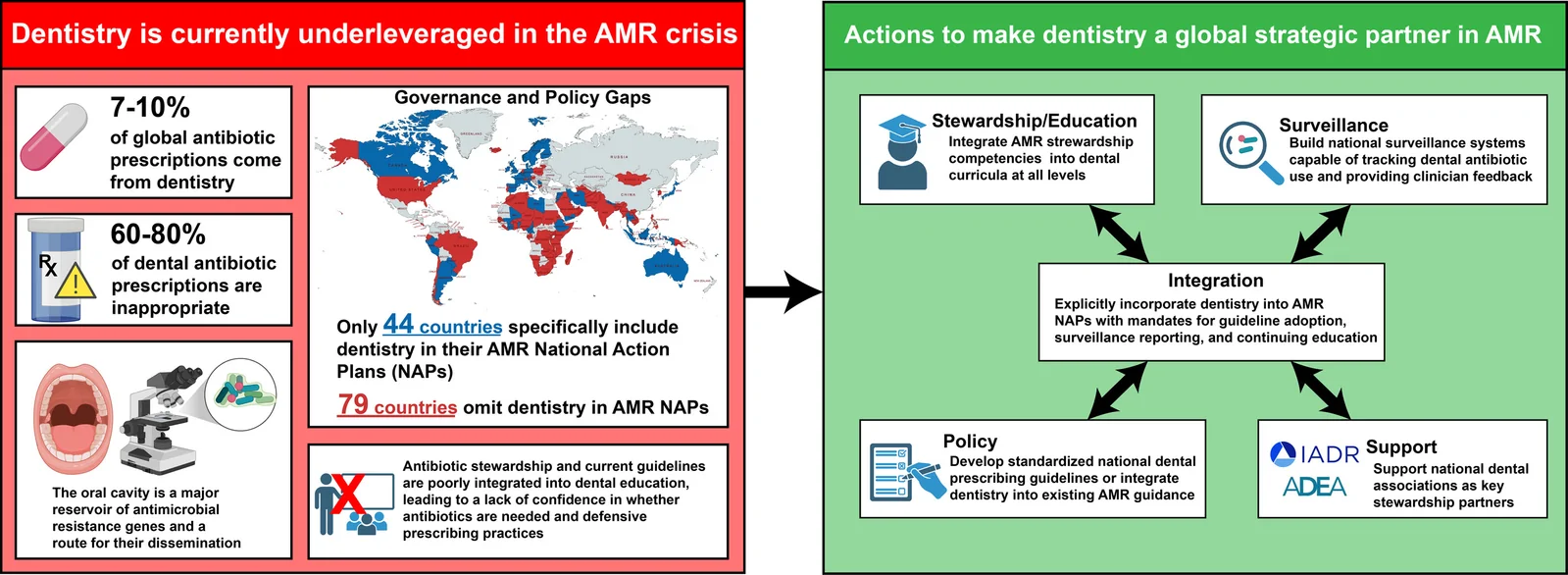
Diagram summarizing why dentistry is currently underleveraged in the AMR crisis and actions suggested to make dentistry a global strategic partner in AMR. Global map illustrating the structural integration or omission of dentistry in national AMR strategies based on a systematic keyword screening of the World Health Organization NAP repository. Blue shading indicates countries whose NAPs explicitly integrate dentistry or dental practitioners (*n* = 44). Red shading highlights countries with an active, searchable NAP that completely omits the dental sector (*n* = 79). Gray shading indicates countries where an active NAP was either unavailable in the WHO archive, unsearchable, or lacked a version translated into standard research languages (*n* = 14).

Despite the clinical and ecological relevance of dentistry to AMR, national and international governance frameworks continue to underrepresent the profession. Reviews of AMR NAPs consistently demonstrate that dentistry is rarely incorporated as a discrete strategic sector (30, 31). Systematic analysis of WHO-archived NAPs performed in preparation for this article found that only 44 countries explicitly mentioned dentistry or dental practitioners, while 79 entirely omitted the sector from their published plans (Fig. 1). Even among countries referencing dentistry, inclusion is superficial, consisting of brief mentions within broader primary care stewardship initiatives without tailored implementation strategies, workforce engagement plans, surveillance metrics, or oral resistome monitoring efforts. Broader governance analyses reinforce this concern with a review of 114 national AMR action plans identifying major weaknesses in accountability and feedback mechanisms (32). These are precisely the types of infrastructure necessary for integrating dental prescribing oversight and stewardship evaluation. Crucially, since national AMR frameworks influence surveillance priorities, funding allocation, implementation research, education, and policy translation, excluding dentistry inevitably fragments global stewardship efforts. The recent United Nations General Assembly declaration, calling for expanded financing and implementation of AMR NAPs, further underscores the importance of explicit inclusion within these frameworks if dentistry is to participate fully in future stewardship and surveillance initiatives (2).

## EVIDENCE THAT STEWARDSHIP INTERVENTIONS WORK

Fortunately, stewardship interventions substantially improve dental antibiotic prescribing. Randomized and quasi-experimental studies show that audit-and-feedback consistently reduces unnecessary prescriptions, while systematic reviews confirm that clinician-targeted tools change prescribing behavior (33). Educational stewardship toolkits have also produced improvements in prescribing appropriateness, including studies in private practice settings where appropriate prescribing increased from 19% to nearly 88% following intervention (34). Similarly, multimodal antimicrobial stewardship programs implemented within academic dental practices have achieved reductions in antibiotic use approaching 73% through a combination of clinician education, guideline development, audit, and feedback (35). National guideline implementation may also shape prescribing patterns at population scale. In Japan, inappropriate prescription of broad-spectrum third-generation cephalosporins at tooth extraction decreased from 58% to 34% over the 3 years following implementation of the AMR NAP (36). Collectively, these findings support the conclusion that dental stewardship is not only necessary but highly achievable when supported by coordinated professional and policy infrastructure.

## EDUCATION, INTEGRATION, AND FUTURE DIRECTIONS

Education and interdisciplinary integration are essential for sustainable dental stewardship. Currently, undergraduate curricula, continuing education efforts, and clinical practices show inadequate coverage of AMR and low awareness of stewardship principles among students (37, 38). Standardizing competency frameworks (39) and implementing continuous professional education are critical steps to integrate dentistry into coordinated public health responses.

Professional organizations and scientific societies, such as the International Association for Dental Research (IADR), the American Dental Education Association (ADEA), and FDI World Dental Federation, can serve as important partners in stewardship implementation, interdisciplinary collaboration, and efforts to integrate dentistry into national and global AMR frameworks (e.g., the CDC Core Elements of Antibiotic Stewardship and the ADA Guidelines on Antibiotic Prophylaxis) (40). Beyond stewardship, dentistry contributes expertise in biofilm biology, microbial ecology, and resistance evolution within complex microbial communities, fields that provide important insights into AMR emergence and dissemination. Greater interdisciplinary integration among dentistry, medicine, pharmacy, microbiology, infection prevention, and public health could improve patient management while reinforcing dentistry’s role within national One Health initiatives.

We propose the following practical policy actions which could help integrate dentistry into national AMR strategies:

1. Explicitly incorporate dentistry into national action plans with mandates for guideline adoption, surveillance reporting, and continuing education.
2. Develop standardized national dental prescribing guidelines or integrate dental sections into existing AMR guidance.
3. Build national surveillance systems capable of tracking dental antibiotic use and providing clinician feedback.
4. Integrate AMR stewardship competencies into dental curricula at all levels.
5. Support national dental associations as key stewardship partners.

Together, these actions could collectively transform dentistry from a peripheral stakeholder into a core strategic partner in global AMR mitigation efforts. As countries continue updating and financing NAPs on AMR, ensuring meaningful inclusion of dentistry will be essential for building more comprehensive and effective responses to the accelerating resistance crisis.
